# Neuroplasticity of Glioma Patients: Brain Structure and Topological Network

**DOI:** 10.3389/fneur.2022.871613

**Published:** 2022-05-13

**Authors:** Kun Lv, Xin Cao, Rong Wang, Peng Du, Junyan Fu, Daoying Geng, Jun Zhang

**Affiliations:** ^1^Department of Radiology, Huashan Hospital, Fudan University, Shanghai, China; ^2^Institute of Functional and Molecular Medical Imaging, Fudan University, Shanghai, China; ^3^Center for Shanghai Intelligent Imaging for Critical Brain Diseases Engineering and Technology Reasearch, Shanghai, China; ^4^Institute of Intelligent Imaging Phenomics, International Human Phenome Institutes (Shanghai), Shanghai, China

**Keywords:** glioma, brain neoplasms, functional connectivity, graph theory, resting-state, connectome, machine learning, neuronal plasticity

## Abstract

Glioma is the most common primary malignant brain tumor in adults. It accounts for about 75% of such tumors and occurs more commonly in men. The incidence rate has been increasing in the past 30 years. Moreover, the 5-year overall survival rate of glioma patients is < 35%. Different locations, grades, and molecular characteristics of gliomas can lead to different behavioral deficits and prognosis, which are closely related to patients' quality of life and associated with neuroplasticity. Some advanced magnetic resonance imaging (MRI) technologies can explore the neuroplasticity of structural, topological, biochemical metabolism, and related mechanisms, which may contribute to the improvement of prognosis and function in glioma patients. In this review, we summarized the studies conducted on structural and topological plasticity of glioma patients through different MRI technologies and discussed future research directions. Previous studies have found that glioma itself and related functional impairments can lead to structural and topological plasticity using multimodal MRI. However, neuroplasticity caused by highly heterogeneous gliomas is not fully understood, and should be further explored through multimodal MRI. In addition, the individualized prediction of functional prognosis of glioma patients from the functional level based on machine learning (ML) is promising. These approaches and the introduction of ML can further shed light on the neuroplasticity and related mechanism of the brain, which will be helpful for management of glioma patients.

## Introduction

Glioma is the most common primary malignant brain tumor in adults, accounting for about 75% of total primary malignant brain tumors. It more commonly occurs in men, and it is more common among White people as compared to other races (1, 2). The incidence rate of CNS malignancy has been increasing in the past 30 years. According to the latest global statistics, China, United States and India are the top three countries with the largest number of cases and deaths of CNS malignant brain tumors (3, 4). The symptoms of gliomas are related to the growth rate and the location of the tumor. There may be systemic symptoms or focal symptoms related to a specific part of the brain.

For example, tumors located in the frontal lobe can cause language disorders; tumors located in the prefrontal lobe, temporal lobe, or corpus callosum usually cause cognition dysfunctions, such as personality changes, mood disorders, and memory deficits (5). These tumors can also result in a wide range of symptoms and signs; 50–80% of patients may have seizures (6, 7), about 30% of patients have headaches, and 15% of patients have symptoms of increased intracranial pressure, such as progressive headaches at night, morning nausea and vomiting, lethargy, and blurred vision caused by papilledema (8). In addition, the prognosis and treatment response are associated with molecular characteristics of gliomas; for example, diffuse glioma with isocitrate dehydrogenase (IDH) 1/2 mutation has a better prognosis than diffuse glioma with IDH wild-type (9, 10). Therefore, the latest World Health Organization (WHO) CNS tumor classification system version (Fifth Edition) further promotes the role of molecular diagnosis in the classification of CNS tumors, along with highlighting and emphasizing the importance of molecular characteristics or genotypes in the comprehensive diagnosis of CNS tumors (11). Considering differences in age, tumor type, and molecular characteristics, the survival period vary greatly; the 5-year overall survival rate is less than 35% (5, 12). Neuroplasticity may preserve neurological function in glioma patients, thereby improving outcomes after resection. Obviously, these symptoms (functional or behavioral deficits) and prognosis are closely related to the neuroplasticity, which are closely related to the patient's quality of life. However, the specific neuroplasticity caused by highly heterogeneous gliomas is not fully understood.

Neuroplasticity is the inherent adaptability in response to changes in the internal environment throughout the course of life, which occurs in the physiological background of growth or learning, and is also an adaptive mechanism that supports functional recovery following brain injury. It allows the continuous processing of short-term, mid-term, and long-term plasticity of neuronal synaptic tissue, which is described as normal plasticity of learning or memory in healthy people, or post-lesional plasticity in patients with traumatic brain injury, stroke, or tumor (13–18). Recently, the concept of Metaplasticity was proposed, it is a higher-order plastic mechanism that regulates the learning rule as a function of the dynamical context, i.e., the initial neural activity that generates metaplasticity before the synaptic plasticity (19). It has been determined that glioma induces large-scale functional plasticity of the peritumoral brain, diseased hemisphere, and partial contralateral hemispheric brain network, homologous to the invaded structure (20–25), included neural reorganization before and after glioma surgery determined by direct electrostimulation mapping (DEM) and transcranial magnetic stimulation (TMS) (26–28). Some advanced technologies in magnetic resonance imaging (MRI) can not only provide a variety of methods for non-invasive detecting neuroplasticity in glioma patients, but also help in assessing improvements in treatment plans (29), such as high-resolution structural T1-weighted imaging (T1WI) and functional magnetic resonance imaging (fMRI), for example, magnetic resonance spectroscopy (MRS) for evaluating biochemical metabolism, diffusion tensor imaging (DTI) that uses fractional anisotropy (FA) to represent quantitative and directional information about water diffusion, and blood oxygenation level-dependent functional magnetic resonance imaging (BOLD-fMRI) (30, 31). At present, neuroplasticity of brain structure and topological network caused by highly heterogeneous gliomas is not fully understood. Therefore, in this article, we review the applications of high-resolution structural T1WI, DTI, and BOLD in assessing brain structure and topological network plasticity in glioma patients, and we further explore future research directions.

## Search Strategy and Selection Criteria

The databases of PubMed, Medline, Scopus, Embase and Web of Science search of the published literature were performed with the combination of the search terms “glioma,” “structure,” “structural,” “plasticity,” “topology,” “topological,” “graph theory,” before and inclusive of April 12, 2022. A total of 1,038 papers were retrieved and 508 duplicates were removed. Subsequently, studies about plasticity of brain cortical structure and topological properties in glioma patients through MRI were included. Meanwhile, other detecting methods rather than MRI, such as magnetoencephalography, DEM, TMS and electrocorticography, and animal-based studies were excluded. In addition, review, case report, abstract, comment and non-English language papers were excluded. Finally, 22 studies met these criteria, and detailed in following neuroplasticity of structure and topological network in glioma patients (Table 1).

**Table 1:** Studies on the neuroplasticity of the structural and topological network in glioma patients.

**References**	**Purpose**	**Sequences/Subjects/Methods**	**Main findings**
Xu et al. (32)	To investigate the structural morphometry of the cortical and subcortical structures in the patients with cerebral gliomas.	High-resolution structural T1WI/13 patients and 14 HCs/SBM	GMV of the right cuneus and the left thalamus significantly increased and positively correlated with the glioma volumes. Structural plasticity might act as the compensation mechanism to better fulfill its functions in patients with cerebral gliomas as the gliomas get larger.
Kinno et al. (33)	To clarify the brain anatomical changes associated with gliomas.	High-resolution structural T1WI/15 patients and 15 HCs/SBM	The left frontal gliomas have global effects on the cortical structure of both hemispheres. The structural changes of the right hemisphere are mainly characterized by the decrease of CT and the slight concomitant decrease of cortical FD, while the structural changes of the surrounding area of gliomas are mainly characterized by the decrease of FD and the relative retention of CT. The structural effects of gliomas, which extend to the distant contralateral regions.
Almairac et al. (34)	To assess the homotopic structural plasticity in case of unilateral glioma of the insula.	High-resolution structural T1WI/84 patients and 24 HCs/VBM	GMV increase in the contralesional insula. Homotopic reorganization that might be a physiologic basis for the high level of functional compensation observed in patients with glioma.
Yuan et al. (39)	To assess the structural and functional plasticity of contralesional MTL in patients with unilateral MTL glioma.	High-resolution structural T1WI and rs-fMRI/68 patients and 40 HCs/VBM and FC	GMV decreased in the contralesional MTL. Intrahemispheric FC decreased between the pHPC and PCC, and positively correlated with cognitive function in both patient groups. The contralesional cortex may have decompensation of structure and function in patients with unilateral glioma, except for compensatory structural and functional adaptations.
Cayuela et al. (40)	To examine cognitive deficits together with brain structural changes in patients with oligodendroglial tumor.	High-resolution structural T1WI/48 patients/SPM8	Patients in Groups 2 and 3 showed significant GM atrophy and more leukoencephalopathy than patients in Group 1. Cognitive deficits were associated with brain atrophy and WM changes. Long-term oligodendroglial tumor survivors who underwent standard RT ± chemotherapy treatment present cognitive impairment, especially in memory and executive functions, associated with late GM and WM damage. Group 1 = 2–5 years, Group 2 = 6–10 years, and Group 3 >10 years
Liu et al. (49)	To investigate the structural and functional plasticity within the CCN in unilateral frontal gliomas.	High-resolution structural T1WI and rs-fMRI/37 patients and 40 HCs/ALFF, ReHo, DC, FC, SPM12	ALFF, GMV and FC were altered in the dmPFC, rSP, lSP in patients with fontal gliomas within the CCN. Increased ALFF in the lSP within the CCN was positively correlated with executive function. Tumors invading the frontal lobe induced contralesional structural and functional reorganization within the posterior CCN in patients with unilateral frontal gliomas. The contralesional superior parietal cortex acts as a functional compensation hub within the CCN, which may protect it against the detrimental effects of tumor invasion on executive functions.
Zhang et al. (50)	To investigate the neuroplasticity of cerebellum and cerebro-cerebellar system induced by left cerebral gliomas that involving language function.	High-resolution structural T1WI and rs-fMRI/78 patients and 44HCs/ALFF, FC, VBM	LGG and HGG patients showed bi-directional brain activity changes in language related cerebellar regions. The brain activity in the increased area was significantly correlated with language and MMSE score. LGG patients showed a larger GMV in the area with enhanced brain activity. The FC between the area with decreased cerebellar activity and the contralateral cerebro-cerebellar system increased in LGG patients.
Yuan et al. (59)	To investigate the structural plasticity and neuronal reaction of the hippocampus in glioma patient presurgery.	High-resolution structural T1WI and rs-fMRI/99 patients and 80 HCs/VBM and dALFF	Remote hippocampal volume increases in LGG and HGG, and a greater response of the ipsilateral hippocampus than the contralesional hippocampus. Bilateral hippocampal dALFF was significantly increased in HGG. The hippocampus has a remarkable degree of plasticity in response to pathological stimulation of glioma. The hippocampal reaction to glioma may be related to tumor malignancy.
Huang et al. (61)	To investigate contralesional compensation in different molecular pathologic subtypes of insular glioma.	High-resolution structural T1WI/52 patients/VBM	Contralesional insula with higher GMV was observed in glioma with IDH mutation. IDH mutation is associated with greater structural compensation in insular glioma.
Xu et al. (71)	To investigate the relationship between possibly altered functional brain network properties and intellectual decline in LGG patients.	rs-fMRI/21 patients/graph theory	The patients displayed disturbed small-world manner (increased *L* and λ) and decreased E_glob_. E_glob_ was positively correlated with IQ test scores in the LGG group. The results provided evidence of reduced E_glob_ for poorer intellectual performance in LGG patients.
Park et al. (72)	To assess functional connectivity in patients with supratentorial brain gliomas with possible alterations in long-distance connectivity and network topology.	rs-fMRI/36 patients and 12 HCs/graph theory	When compared with HCs, the patients showed decreased long distance, inter-hemispheric connectivity, increased local efficiency, but global efficiency, clustering coefficient, and small-world topology were relatively preserved.
Fang et al. (73)	To investigate changes in the FC and topological properties of the insular lobe in patients with LGG.	rs-fMRI/35 patients and 33 HCs/graph theory	The nodal shortest path length of the right insular lobe was significantly increased in the insL group compared to the control group. Additionally, FC was increased in the functional edges originating from the left insular lobe in the insR group compared to the control group. The contralesional insular lobe is crucial for network alterations. The detailed patterns of network alterations were different depending on the affected hemisphere. The observed network alterations might be associated with functional network reorganization and functional compensation.
Baene et al. (75)	To examine whether the functional global organization of the undamaged regions differs and specific network topology features of the undamaged areas between LGG and HGG patients.	rs-fMRI/40 LGG and 40 HGG patients/graph theory	LGG patients showed lower contralesional intramodular connectivity, lower contralesional ratio between intra- and intermodular connectivity, and greater contralesional intermodular connectivity than HGG patients. In the hemisphere contralateral to the lesion, there is a lower capacity for local, specialized information processing coupled to a higher capacity for distributed information processing in LGG patients.
Hart et al. (76)	To apply connectome analysis in patients with brain tumors to characterize overall network topology and individual patterns of connectivity alterations.	rs-fMRI/5 patients/ graph theory	These patients retained key characteristics of complex networks in HCs, including ubiquitous small-world organization. The robustness of general networks to damage was predicted, but accompanied by the disproportionate vulnerability of a core of hubs. The tumor produced a continuous reduction of local and long-range connectivity, and different connection loss patterns depend on the location of the lesion.
Huang et al. (77)	To detect differences in the whole brain topology among LGG patients before and after operation.	rs-fMRI/12 patients and 12 HCs/graph theory	Preoperative and postoperative LGG groups showed disturbed networks and widely spread in the strength and spatial organization of brain networks, which negatively related to worse MoCA scores. It is considered that the changed small-world network may be the cause of cognitive dysfunction in patients with frontal LGG.
Tao et al. (78)	To analyze the topological properties of brain structural networks, and discuss the function compensatory mechanism of LGG patient.	DTI/20 patients and 20 HCs/graph theory	LGG patients showed altered topological metrics with global parameters and betweenness centrality values of hub nodes. These alterations may be a compensatory mechanism in LGG patients to adapt to cognitive requirements.
Liu et al. (79)	To reveal the rich-club organization and topological patterns of WM structural networks associated with cognitive impairments in patients with frontal and temporal gliomas.	DTI/31 patients and 14 HCs/graph theory	Both FTumor and TTumor showed altered local network efficiency and deficits in the nodal shortest path in the left Rolandic operculum and DC of the right dorsolateral and SFGmed. TTumor patients showed a significantly higher DC in the right dorsolateral and SFGmed, a higher level of betweenness in the right SFGmed, and higher nodal efficiency in the left middle frontal gyrus and right SFGmed than FTumor patients. Rich-club organization was disrupted, with increased structural connectivity among rich-club nodes and reduced structural connectivity among peripheral nodes in FTumor and TTumor patients. Altered local efficiency in TTumor correlated with memory function, while altered local efficiency in FTumor correlated with the information processing speed.
Fang et al. (80)	To delineate topological networks and investigate characteristics of functional networks in patients with GRE.	rs-fMRI/30 patients and 20 HCs/ graph theory	Temporal lobe glioma and GRE changed the visual network. The changes in the visual network caused by GRE are different to those caused by glioma itself. These findings provide new insights into the changes in the brain network caused by GRE.
Fang et al. (81)	To investigate the alterations of neural networks in temporal LGG patients with GRE.	rs-fMRI/56 patients and 28HCs/graph theory	The shortest path length, clustering coefficient, local efficiency, and vulnerability were greater in the non-GRE group than in the other groups. The nodal efficiencies of two nodes (mirror areas to Broca and Wernicke) were weaker in the non-GRE group than in the other groups. The node of DC (Broca), nodal local efficiency (Wernicke), and nodal clustering coefficient (temporal polar) were greater in the non-GRE group than in the HCs. Temporal lobe gliomas in the right hemisphere altered the language network. Glioma itself and GRE altered the network in opposing ways in patients with right temporal glioma.
Fang et al. (82)	To investigate alterations of functional networks in patients with prefrontal glioma and GRE	rs-fMRI/65 patients and 25HCs/graph theory	The reduction of FC between the medial BA 6 and caudal ventrolateral BA 6 in the ipsilateral hemisphere and the shortening of the path length of the sensorimotor network were characteristics alterations in prefrontal gliomas patients with GRE onset.
Zhou et al. (83)	To identify alterations to the subcortical brain networks caused by glioma and GRE.	DTI/61 patients and 14 HCs/graph theory	Compared with HCs, the epilepsy groups showed raletively intact WM networks, while the non-epileptsy groups had damaged network with lower efficiency and longer path length.
Yang et al. (84)	To discuss the mechanism of ISE using DTI-based graph theoretical analysis.	DTI/20 patients and 10 HCs/graph theory	The connections between A123truL and A4ulL, A123truR and A4tR, and A6mL and A6mR were significantly decreased in the epilepsy group. The global efficiency of the epilepsy group decreases significantly, while the shortest path length increases. Disconnection of the hub nodes A6mL and A6mR in ISE patients results in a subsequent decrease in global and local network efficiency.

*A123truL, Brodmann area 1,2,3, left body trunk; A123truR, Brodmann area 1,2,3, right body trunk; A4tR, Brodmann area 4, right body trunk; A4ulL, Brodmann area 4, left upper limb; A6mL, left medial area 6; A6mR, right medial area 6; BA, Brodmann area; CCN, cognitive control network; CT, cortical thickness; dALFF, dynamic amplitude of low-frequency fluctuation; DC, Degree Centrality; dmPFC, dorsal medial prefrontal cortex; DTI, Diffusion tensor imaging; E_glob_, global efficiency; FC, functional connectivity; FD, fractal dimension; FTumor, frontal tumors; GM, gray matter; GMV, GM volume; GRE, glioma-related epilepsy; HCs, healthy controls; HGG, High-grade glioma; IDH, isocitrate dehydrogenase; IQ, intelligence quotient; ISE, intraoperative stimulation related epilepsy; L, characteristic path length; LGG, Low-grade glioma; lSP, left superior parietal cortex; MoCA, Montreal Cognitive Assessment; MMSE, Mini Mental State Examination; MTL, medial temporal lobe; PCC, posterior cingulate cortex; pHPC, posterior hippocampus; ReHo, Regional Homogeneity; rs-fMRI, resting-state functional Magnetic resonance imaging; rSP, right superior parietal cortex; RT, radiation therapy; SBM, surface-based morphometry; SFGmed, medial superior frontal gyrus; SPM, Statistical Parametric Mapping; T1WI, T1-weighted imaging; TTumor, temporal tumors; VBM, voxel-based morphometry; WM, White matter; λ, normalized characteristic path length*.

## Neuroplasticity of Structure in Glioma Patients

Post-lesional plasticity is determined by brain network functional plasticity in glioma patients. Although several non-invasive functional neuroimaging methods have extensively demonstrated the mechanism of this functional redistribution in glioma patients, less attention has been paid to the structural plasticity of the cortex and subcortical structures related to the volume of glioma. To determine whether there is cortical and subcortical plasticity in patients with gliomas and to examine whether subcortical and contralateral cortical structures can be actively reorganized, Xu et al. (32) compared the differences in cortical and subcortical gray matter (GM) volume (GMV) in 13 patients with different grades of gliomas in the left hemisphere with that of 14 healthy controls (HCs) based on surface-based morphometry (SBM). The study found that the GMV of the right cuneus and left thalamus of patients with glioma increased significantly compared with that of the HCs. The GMV of these areas was positively correlated with the glioma volume. In addition, with the enlargement of glioma, structural plasticity, as a compensation mechanism, may better fulfill its functions in glioma patients. To clarify the brain anatomical changes associated with gliomas, Kinno et al. (33) compared the cortical structures of 15 patients with left frontal gliomas and 15 HCs through SBM. The study found that the left frontal gliomas have global effects on the cortical structure of both hemispheres. The structural changes of the right hemisphere are mainly characterized by the decrease of cortical thickness (CT) and the slight concomitant decrease of cortical fractal dimension (FD), while the structural changes of the surrounding area of gliomas are mainly characterized by the decrease of FD and the relative retention of CT. These results illustrate the structural effects of gliomas extending to the distant contralateral regions. Based on voxel-based morphometry (VBM), Almairac et al. (34) found that the volume of contralateral GM increased in insular glioma. Although studies have shown that homologous plasticity may be the physiological basis of high-level functional compensation observed in glioma patients, whether this plasticity model can be extended to other cortical regions and if it can be extended to other types of brain injuries, such as stroke or traumatic brain injury, remains to be further studied.

The maintenance of cognitive function in patients with glioma is related to the plasticity of the morphology and function of nervous tissues of the CNS (17, 35–38). Yuan et al. (39) compared 33 patients with left and 35 patients with right medial temporal lobe (MTL) gliomas and 40 HCs; they found that the GMV of the contralateral MTL decreased, and intra-hemispheric functional connectivity (FC) decreased between the posterior hippocampus (pHPC) and posterior cingulate cortex (PCC). In the two groups of patients, the intra-hemispheric FC between the pHPC and PCC was positively correlated with cognitive function. The study showed that in addition to structural and functional adaptive compensation, there may be structural and functional decompensation in the contralateral cortex of patients with unilateral glioma. Another glioma study related to cognitive function observed the changes in cognitive impairment and brain structure in long-term oligodendroglioma patients receiving radiotherapy (RT) and chemotherapy. In the study, Cayuela et al. (40) divided 48 patients with oligodendroglioma who received RT ± chemotherapy into three groups according to the treatment years: group 1, treated for 2–5 years; group 2, treated for 6–10 years; and group 3, treated for more than 10 years. The study found that patients in groups 2 and 3 showed significant GM atrophy and more leukoencephalopathy than patients in group 1, and cognitive dysfunction was related to brain atrophy and WM changes. However, due to the retrospective nature of the study, the study could not determine whether there are longitudinal cognitive changes caused by chemotherapy and/or RT. Furthermore, the contribution of RT and chemotherapy toward cognitive impairment could not be ruled out. In addition, the cognitive control network (CCN) is a frontal-parietal circuit that participates in top-down, attention-dependent executive functions, such as decision-making and task switching (41–43). It is composed of the main brain regions of the dorsomedial prefrontal cortex, bilateral prefrontal cortex, and bilateral superior parietal cortex (44–48). In the frontal lobe involved in the executive function of the CCN, Liu et al. (49) paid special attention to the influence of the contralateral remote brain region of the CCN damaged area on the maintenance of cognitive function in injury-induced structural and functional neuroplasticity. They speculated that there is a functional compensation center in the CCN in patients with unilateral frontal gliomas, which can protect them from the adverse effects of tumor invasion on executive functions. They compared 16 patients with left frontal gliomas, 21 patients with right frontal gliomas, and 40 HCs, and calculated GMV and relevant functional indicators, including Regional Homogeneity (ReHo), amplitude of low-frequency fluctuation (ALFF), degree centrality (DC), and FC. The study found that as compared with HCs, when the tumor invaded the left frontal lobe, the ALFF of the dorsomedial prefrontal cortex (dmPFC) decreased in the patients' CCN, while the ALFF of the right supraparietal cortex (rSP) increased; when the tumor invaded the right frontal lobe, the GMV and ALFF of the left supraparietal cortex (lSP) in the patients' CCN increased significantly. Moreover, the FC between the lSP and dmPFC and between the lSP and rSP within the patients' CCN increased significantly. The increased ALFF in the lSP was positively correlated with executive functions. This study shows that patients with unilateral frontal gliomas can develop contralateral structural and functional reorganization in the CCN, and the contralateral parietal cortex plays a role in the functional compensation center in the CCN, which can protect the CCN from the adverse effects of tumor invasion on executive function. Meanwhile, in the neuroplasticity of cerebellum and cerebro-cerebellar system induced by left cerebral gliomas that involving language function, Zhang et al. (50) compared brain activity, FC and GMV of 46 patients with LGG, 32 with HGG and 44 HCs. The study found that LGG and HGG patients showed bi-directional brain activity changes in language related cerebellar regions. The brain activity in the increased area was significantly correlated with language and Mini Mental State Examination (MMSE) scores. Structurally, LGG patients showed a larger GMV in the area with increased brain activity, indicating the change of structure function coupling in the cerebellum. The functional connection between the area with decreased cerebellar activity and the contralateral cerebro-cerebellar system increased in LGG patients. This study provides evidence for the important role of cerebellum and cerebro-cerebellar system in neural plasticity after brain language network injury.

The hippocampus is one of the few areas in the adult brain that contain neural stem cells. During the lifetime of humans and animals, the hippocampus can continuously produce neurons (51–54). Neuroimaging studies have shown that the hippocampus has significant plasticity to various training, experience, and mental disorders. For example, the size of the human hippocampus has been shown to increase with aerobic exercise and will return to baseline levels after 6 weeks without aerobic exercise (55). The change of hippocampal volume is considered to be a potentially useful biomarker for the development or treatment of major depressive disorder. The greater the number of depressive episodes, the greater the loss of hippocampal volume. Antidepressant therapy or electroconvulsive therapy can increase hippocampal volume (56–58). Accordingly, Yuan et al. (59) evaluated the structural plasticity and neuronal response of the hippocampus in patients with glioma. The study compared hippocampal volume and dynamic ALFF (dALFF) of 52 patients with low-grade gliomas (LGGs), 47 patients with high-grade gliomas (HGGs), and 80 HCs. The study found that the volume of the distal hippocampus increased in patients with LGGs and HGGs, and the hippocampus on the affected side had a greater response than the contralateral hippocampus. Bilateral hippocampal dALFF was significantly increased in patients with HGGs. The study also showed that the hippocampus has significant plasticity to the pathological stimulation of glioma, and the response of the hippocampus to glioma may be related to the degree of tumor malignancy. However, the study lacked histological evidence to explain the physiological mechanism of increase in hippocampal volume in patients with glioma.

The cortical plasticity pattern of gliomas is hierarchical (60). This hierarchical pattern is particularly suitable for LGGs because they show the characteristics of slow growth and low invasiveness, which leaves enough time for the process of plasticity (61). It should be noted that based on the 2016 version, the 2021 version of the WHO CNS tumor classification system (Fifth Edition) further promotes the importance of molecular diagnosis in the comprehensive diagnosis of CNS tumors, which is highly correlated with tumor aggressiveness and prognosis (11, 62, 63). There is evidence that IDH wild-type LGGs should be considered as glioblastomas (grade IV) because they have similar clinical and genetic characteristics (64). Therefore, different molecular subtypes may reflect different biological behaviors and may lead to different results of neuroplasticity. Based on this, a recent study compared the differences in the structural plasticity of the contralateral side of different molecular subtypes. Huang et al. (61) compared 52 patients with insular glioma based on the tissue grade (low-grade and high-grade) and molecular pathology (IDH mutation, telomerase reverse-transcriptase promoter mutation, and 1p19q coding deletion). This study showed that 320 voxel clusters were observed in the contralateral insula of the IDH mutant type, and the GMV of the entire insula was also larger than that of the IDH wild-type. However, whether there is an association between molecular pathological subtypes and cortical structural reorganization in gliomas other than in insula gliomas requires further study.

## Neuroplasticity of Topological Network in Glioma Patients

Graph theory is a branch of mathematics that studies the formal description and analysis of graphs. A graph is simply defined as a set of nodes (vertices) linked by connections (edges). In the neural field, a node can be a brain region or network, and an edge is a functional connection between brain regions or networks. This connection (edge) can be directed or undirected. Graph theory can also give weights to the connection (edge) to represent the relevant size, such as the time or distance between nodes. Based on graph theory, quantitative analysis of complex networks can be performed, including robustness of networks, for example, random, small-world, scale-free, hierarchical, and geometrical networks (65). The brain network is composed of spatially distributed, but interconnected, brain regions, with the characteristics of a complex network. Many studies have shown that the connection pattern of the entire brain functional network exhibits effective small-world properties with high clustering coefficient and low path length (66), allowing local separation and global integration of information (67–70). Topological changes of brain networks in glioma patients can be explored by calculating relevant network property metrics, such as average path length, clustering coefficient, global efficiency (E_glob_), local efficiency, node degree, betweenness, and node efficiency (Table 2).

**Table 2:** Main network metrics of graph theory.

**Metrics**	**Definition/meaning**
Average path length	The average of the distances of all point pairs in the network.
Clustering coefficient	The ratio of the number of edges between a node and its neighbors to the maximum possible number of edges.
Global efficiency	Measure the information transmission capacity between nodes in the network.
Local efficiency	The global efficiency of the sub-network formed by the interaction of each neighbor.
Node degree	The number of connections that link it to the rest of the network.
Betweenness	The contribution of each node to the shortest path between all other pairs of points.
Nodal efficiency	Information transmission capacity of each node in the network.
Robustness	The ability of a network to maintain its typical network characteristics after removal of specific node(s) or edge(s).

Although the average survival time of patients with LGG may be extended to more than 10 years, many LGG survivors suffer from devastating mental disorders (such as intellectual decline) that have a negative impact on their quality of life. Intellectual decline of glioma patients is related to many factors, such as the tumor itself, tumor-related epilepsy, anti-epileptic drugs, and tumor-related treatment. Considering the graph theory analysis based on resting state functional MRI (rs-fMRI), Xu et al. (71) explored the relationship between brain network characteristics and intellectual performance of LGG patients. They compared 12 patients with LGG in the left hemisphere and 9 patients with LGG in the right hemisphere. The study found that LGG patients showed disorder of small-world properties, manifested by the increase of the characteristic path length (*L*), the normalized characteristic path length (λ), and the decrease in E_glob_. In addition, E_glob_ in the LGG group was positively correlated with intelligence quotient test scores. This study provides evidence of changes in functional network properties for the mechanism of intellectual impairment in LGG patients.

Focal brain lesions can lead to morphological and functional changes in remote brain regions, such as the contralateral brain area at the homologous level of the lesion. Meanwhile, Park et al. (72) and Fang et al. (73) determined that focal gliomas can cause altered interhemispheric connectivity and altered local topological properties of the contralateral hemisphere. In addition, when the disease gradually progresses, functional compensation and structural plasticity of these areas seem to be more effective. However, it is not clear whether the momentum of the development of the disease also modulates the functional network topology of the remote brain area. Compared with HGGs (WHO III–IV), LGGs (WHO I–II) tend to grow slower, are less invasive, and have a lower degree of cell infiltration and proliferation. In contrast, HGGs, especially grade IV HGGs, grow much faster. Studies have shown that there is a 10-fold difference in the growth rate, wherein LGG grows about 4 mm/year, while HGG grows about 3 mm/month (74). This difference in the growth rate of HGG and LGG provides the best model for the study of this problem. De et al. (75) included 40 LGG patients and 40 HGG patients for a graph theory analysis based on rs-fMRI. The study found that LGG patients showed lower intra module connectivity and ratio between intra module and inter module connectivity and greater inter module connectivity than HGG patients. Compared with HGG patients, LGG patients had lower separation and higher integration in the contralateral hemisphere. This provides some evidence that brain lesions regulate the functional network topology of remote brain regions.

Gliomas are thought to induce multiple modes of cognitive impairment, and are widely considered to be related to changes in the functional network topology; however, the ways in which the tumor affects cognitive functions in the form of neural networks remains unknown. Hart et al. (76) collected rs-fMRI data from five patients with right temporal–parietal–occipital glioblastoma with the aim of applying connectome analysis to characterize overall network topology and individual patterns of connectivity alterations in brain tumor patients. The study found that these patients retained key characteristics of complex networks in HCs, including ubiquitous small-world organization, and the robustness of general networks to damage was predicted, but accompanied by the disproportionate vulnerability of a core of hubs. The tumor produced a continuous reduction of local and long-range connectivity, and different connection loss patterns depend on the location of the lesion. The study suggests that in the future, people can use such data to model functional plasticity and recovery of cognitive deficits. Brain tumor patients are often associated with preoperative small-world configuration and loss of neurocognitive function. Huang et al. (77) collected rs-fMRI data of 12 patients with frontal lobe LGG before and after surgery and compared with 12 HCs, and applied Montreal Cognitive Assessment (MoCA). The study found disturbed networks in both LGG groups and widely spread in the strength and spatial organization of brain networks, and negatively related to worse MoCA scores. It is considered that the changed small-world network may be the cause of cognitive dysfunction in patients with frontal LGG. However, Tao et al. (78) believes that the abnormal topological properties of LGG patients may be a compensatory mechanism to adapt to cognitive requirements.

The brain WM is thought to form the basis of brain function. Previously, some studies focused on the functional disruption of the connectome that occurred in glioma patients, rather than on anatomical networks. “Functional connection” usually reflects the temporal correlation between fMRI time series from remote brain regions in space, while “structural connection” is related to WM fiber bundles. It is unclear whether gliomas cause changes related to cognitive impairment in the WM structural network describing anatomical connections. Liu et al. (79) used DTI-based graph theory analysis to compare 13 patients with frontal lobe and 18 patients with temporal lobe glioma with 14 HCs, aiming to reveal the organization and topological pattern of Rich-Club in the brain WM structure network of patients with frontal and temporal gliomas. The study found that, as compared to the HCs, in the patients, frontal lobe and temporal lobe tumors did not change significantly in terms of small-world properties and global network efficiency; however, the local network efficiency did change. Secondly, patients with frontal and temporal lobe tumors showed similar deficits in the nodal shortest path of the left Rolandic operculum and the DC of the right dorsolateral and medial superior frontal gyrus (SFGmed). Third, compared to patients with frontal lobe tumors, patients with temporal lobe tumors had significantly higher DCs on the right dorsolateral and SFGmed, a higher betweenness in the right SFGmed, and higher nodal efficiency in the left middle frontal gyrus and the right SFGmed. In addition, the Rich-Club organization of patients with frontal lobe tumors and temporal lobe tumors was destroyed, with increased structural connectivity between Rich-Club nodes, while the structural connectivity between peripheral nodes decreased. Changes in local efficiency of temporal lobe tumors were related to memory function, while changes in local efficiency of frontal lobe tumors were related to information processing speed. This study shows that the compensatory mechanism plays a key role in the global topology formation of patients with frontal and temporal lobe tumors.

Temporal and frontal lobe gliomas are often accompanied by epilepsy. Previous studies only focused on the relationship between primary seizures and changes in functional networks. However, the changes in the functional network caused by glioma-related epilepsy (GRE) are also affected by the glioma and epilepsy. Therefore, the previous conclusions on the changes in functional network in primary epilepsy are insufficient and hinder appropriate preoperative prevention and intraoperative treatment. In addition, the changes in cerebral network caused by glioma and GRE are not completely clear. Around frontal and temporal GRE and in regard to graph theory analysis based on rs-fMRI, Fang et al. found that both frontal lobe and temporal lobe GRE will cause changes in the topological properties of brain functions, and temporal lobe GRE can cause changes in the topological properties of visual networks (80) and language networks (81); prefrontal lobe GRE can cause changes in the topological properties of sensorimotor networks (82). Meanwhile, Zhou et al. (83) identified altered topological properties of epilepsy-related white matter network in patients with frontal glioma. Furthermore, awake craniotomy with intraoperative stimulation has been used for glioma surgical resection to preserve quality of life, where epilepsy may occur in 5–20% of cases with serious consequences. Yang et al. (84) recruited 20 patients with motor-area glioma, divided into two groups (epilepsy and non-epilepsy) based on the presence of intraoperative stimulation related epilepsy (ISE), vs. 10 HCs with aims to explore the mechanism of ISE through DTI-based graph theory analysis. The study found that the epilepsy group had abnormal topological properties such as a significant decrease in connection and global efficiency, and an increase in the length of the shortest path. This study argues that local disconnection of hub nodes in ISE patients, resulting in subsequent lower efficiency of global and local network, and should be treated with caution when hub nodes are involved during intraoperative electrical stimulation.

## Future Directions

### High-Resolution Structural T1WI and Graph Theory in Glioma Patients

Brain is a very complex and delicate organ. Previous studies on the structure plasticity based on high-resolution structural T1WI and plasticity of structural and functional topological networks based on graph theory provide some evidence for the neuroplasticity of glioma patients. However, the plasticity caused by gliomas involving specific brain regions (such as the hippocampus, insula, Broca), different molecular subtypes (such as IDH mutation and wild-type), different grades (such as LGG and HGG), and accompanied by neurological deficits is still not fully understood. In addition, whether there is a structural covariance relationship between these brain regions with structural or topological changes needs to be further evaluated. Moreover, the brain mechanism involved in the improvement or deterioration of neurological deficits before and after treatment (such as surgery, radiotherapy, and chemotherapy) in patients with different rehabilitation training strategies requires more research. Meanwhile, some non-MRI methods have also shown neuroplasticity caused by brain tumor, such as TMS, DEM. Therefore, the integration of the above MRI methods and non-MRI methods may lead to some new findings. Furthermore, in future studies, it is necessary to improve the homogeneity of the study population, along with increasing the sample size.

### MRS Explores the Metabolism of Compensatory Brain Area in Glioma Patients

MRS is a quantitative method that analyzes specific nuclei and their compounds by using nuclear magnetic resonance phenomenon and chemical shift. It can non-invasively study the metabolism, along with physiological and biochemical changes of internal human organs and tissues (85, 86). Hydrogen proton (^1^H) MRS is the most commonly used technique and can be performed under long echo time (288 ms or 144 ms) and short echo time (35 ms). Regional biochemical differences can be better evaluated using single voxel technique for the ROI or a multi-voxel technique covering a wider region (87). Numerous studies (88–90) have shown that ^1^H-MRS can quantitatively or semi-quantitatively detect brain metabolites, including glutamate (Glu), glutamine (Gln), γ-aminobutyric acid (GABA), N-acetylaspartate (NAA), creatine and phosphocreatine (tCr), myo-inositol (mIns), choline (Cho), and glycerophosphorylcholine (GPC), and provide biochemical information such as brain nerve cell activity, energy metabolism, cell density, cell membrane transport, anaerobic metabolism, neurotransmitter content, and cell membrane permeability.

**Figure 1:**
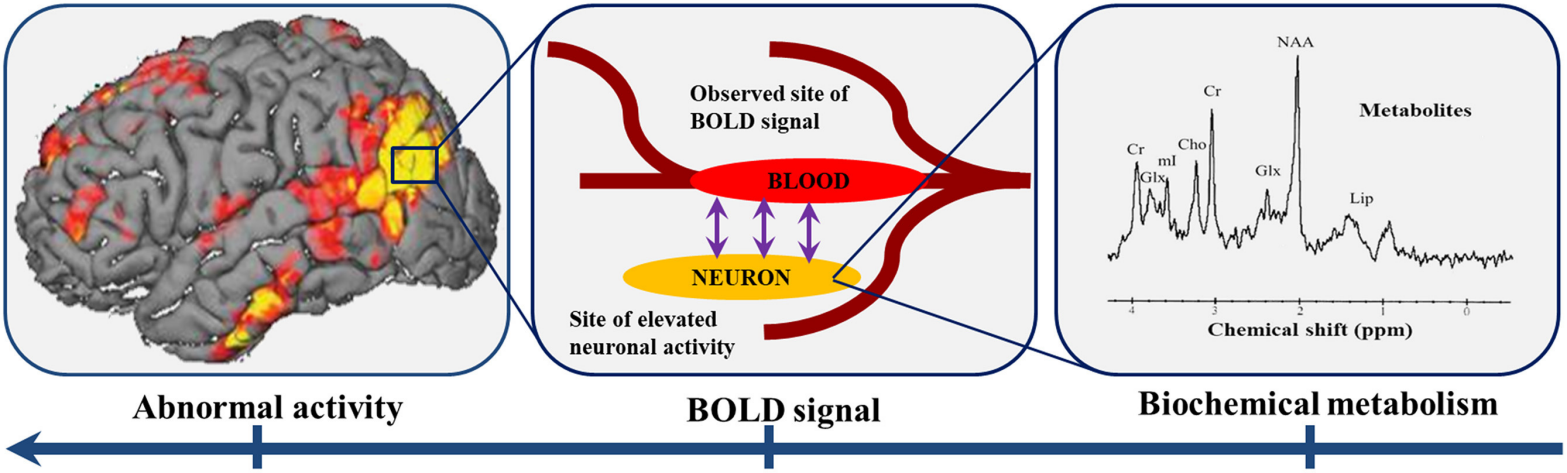
Neurotransmitter-mediated signal transmission plays an important role in regulating cerebral blood flow. The abnormal BOLD signal in the brain region reflects the abnormal activity of neurons in the corresponding region, which may be accompanied by changes in the biochemical metabolism of the brain. BOLD: blood oxygen saturation level dependent.

*In vivo* MRS has been widely and effectively applied to many craniocerebral diseases, such as tumors, ischemia, infection, epilepsy, metabolic diseases, dementia, and mental disorders. MRS is a highly valuable application in the diagnosis, grading, and follow-up after treatment of brain tumors as it analyzes the relative or absolute concentration of neurometabolites in brain tissues (91, 92). In addition, studies (93, 94) have shown that cerebral blood flow is regulated by neurons and astrocytes; neurotransmitter-mediated signal transmission plays an important role in regulating cerebral blood flow, which is mainly mediated and controlled by astrocytes (94). Thus, changes in cerebral blood supply can be accompanied by changes in cerebral neurotransmitters (Figure 1). Previous studies have confirmed that there are cerebral structure and functional changes in the glioma patients, which may closely relate to brain metabolism. Therefore, further studies are needed to determine whether neurotransmitter or neurometabolite changes are also present in the structural or functionally compensated brain regions of patients with gliomas. This may reflect, to some extent, the metaplasticity of gliomas, that is, the initial neural activity before neuroplasticity.

### Connectome-Based Lesion Network Mapping in Glioma Patients

The traditional localization method of brain lesions is to identify the overlapping parts of lesion locations in patients with similar symptoms by connecting neurological symptoms with specific brain regions. Although this method is powerful and has been widely applied, its use is limited when the symptoms do not belong to a single area or originate from the dysfunction of the area connected to the lesion rather than the lesion itself (95). For example, many lesions that cause language disorders are located outside the left frontal cortex (96, 97), many lesions that cause memory damage are located outside the hippocampus (98), and lesions that disrupt social behavior usually occur outside the frontal lobe (99). Therefore, the relationship between symptoms and lesions is sometimes not straightforward. Secondly, the location of symptoms based on lesions is also restricted by the occurrence of many complex symptoms in patients without obvious brain lesions. Common neurobehavioral and psychiatric diseases, such as delirium, amnesia, autism, and schizophrenia, occur in patients without obvious brain disease. An increasing number of studies have found that many neurological and psychiatric symptoms are closer to being supported by network connections in different areas of the brain (100).

**Figure 2:**
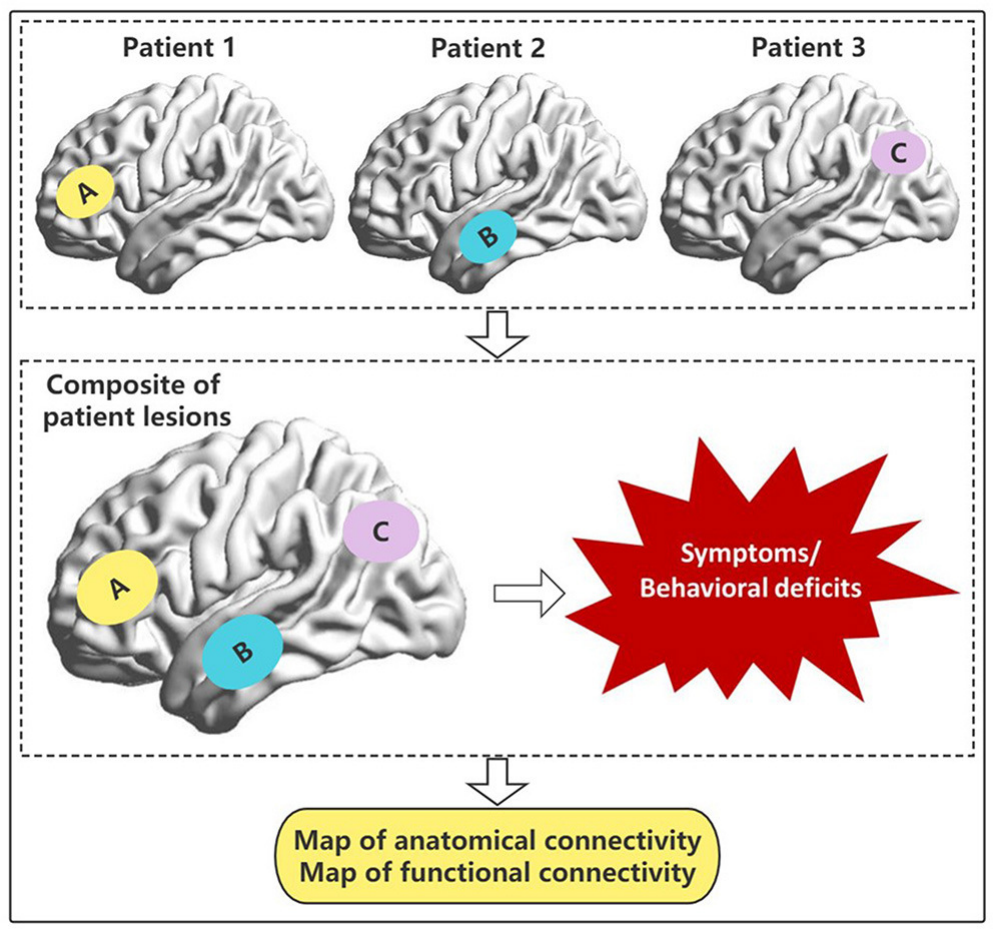
Lesions or destruction of patients in different brain regions or networks **(A–C)** leads to similar symptoms or behavioral deficits. Based on the connectome, it is possible to explore whether there are common anatomical and/or functional connected brain regions.

The distribution of functional areas of the brain is a highly complex network; each part is relatively independent and highly unified. All functions are the result of interactions within this huge network, that is, connectome. Through the connectome, lesions in different parts of the brain that cause the same symptoms can be connected to a common network in a way that was previously impossible (Figure 2). This method is called lesion network mapping and has been applied to a variety of neuropsychiatric symptoms, many of which have deviated from the traditional lesion location methods. These symptoms include auditory hallucinations, aphasia, pain, hemiplegia, Parkinson's disease, and behavioral disorders (100). In each case, lesions at different sites causing the same symptoms are part of a single brain network, defined by their FC. This is an improvement in the traditional lesion location analysis, because the same symptoms are often caused by the above-mentioned connectivity, disconnection, and diaschisis in different locations of the lesion (101–104). The connectome localization may provide new findings for lesion network mapping of glioma patients, such as the mapping of diseased brain regions or networks causing language impairment in different parts outside the frontal lobe, which will make it possible to formulate more accurate pre-treatment plans for patients with complex neurological and psychiatric symptoms. These plans include discovering new therapeutic targets or avoiding damage to related brain areas that have compensatory or auxiliary functions.

Using the connectome, behavioral deficits can be predicted according to the location of the brain lesion and its associated network or brain regions. This is achieved by embedding the patient's lesion into the functional and structural connection map of healthy subjects, and obtaining the set of structural and functional connections passing through the lesion, so as to indirectly estimate its impact on the whole brain connectome. In order to verify the clinical relevance of these methods, Salvalaggio et al. (105) quantified the prediction of behavioral deficits in 132 first-time stroke patients in a prospective cohort study at 2 weeks post-injury, which linked deficits in multiple functional areas (left and right visual, left and right motor, language, spatial attention, spatial and verbal memory) with lesion patterns and indirect structural or functional disconnection. Lesions and indirect structural disconnection can predict behavioral deficits in all areas except language and memory. Although indirect functional disconnection can predict right visual field defect, it cannot predict behavioral deficits, especially cognitive impairment. The study also showed that indirect structural assessment successfully predicted behavioral deficits after stroke, and its level was equivalent to the lesion pattern. This technique may therefore provide some ideas for the prediction of behavioral or functional deficits before and after surgery, based on the different locations of gliomas.

### Model Establishment and Prediction Based on Machine Learning

Currently, the prediction models of glioma based on machine learning (ML) mostly focus on the anatomical structure and morphology (106–109), and there are few reports at the functional level. In addition, previous studies involving the functional level mostly adopted a research strategy based on the comparison between case-control groups; that is, a group of patients and a group of comparable individuals who do not suffer from the disease, considered as controls, that were compared using a specific imaging index to judge whether statistically significant imaging differences could be obtained. These findings only represent the differences between groups. It may not be a suitable detection method for individuals with subtle pathological differences. ML connects statistics and computer science, so as to develop algorithms to improve performance by contacting meaningful data rather than clear instructions (110), which can be used for functional change prediction and individualized recognition of different glioma patients. However, with the rapid development and popularization of ML methods, though researchers are paying attention to the improvement of prediction ability, they are far from the biological understanding of the research results. These disconnect between predictive models and biological significance will inherently limit a wide range of clinical transformations. Therefore, it is necessary to introduce biological significance into the field of ML through various available biological methods, such as genome association, immunohistochemical analysis, local micro pathological image texture, and macro histopathological marker expression (111), especially among patients with highly heterogeneous gliomas with different grades and types which are closely related to treatment and prognosis.

## Conclusion

Previous studies have found that glioma itself and related functional impairments, such as cognition and epilepsy, can lead to neuroplasticity of structure and topological networks, using high-resolution structural T1WI, DTI and rs-fMRI. These MRI technologies can continue to be further used to explore the neuroplasticity of the structure and topological networks of highly heterogeneous gliomas. MRS and connectome provide powerful methods for detecting biochemical alterations and abnormal networks mapping outside of lesion, respectively. In addition, the individualized prediction of functional prognosis of glioma patients from the functional level based on ML is promising. Overall, these approaches can further shed light on the neuroplasticity and related mechanism of the brain, which will be helpful for management of glioma patients.

## Author Contributions

DG and JZ designed the outline for the study. KL, XC, and RW acquired the data. KL wrote the paper. PD and JF offered assistance to correct syntax errors in the paper. All authors contributed to this article and approved the final version.

## Funding

This work was supported by the Clinical Research Plan of SHDC (Grant Number: SHDC2020CR3020A), Research Startup Fund of Huashan Hospital Fudan University (Grant Number: 2021QD035), Shanghai Sailing Program (Grant Number: 22YF1405000), and Greater Bay Area Institute of Precision Medicine (Guangzhou) (Grant Number: KCH2310094).

## Conflict of Interest

The authors declare that the research was conducted in the absence of any commercial or financial relationships that could be construed as a potential conflict of interest.

## Publisher's Note

All claims expressed in this article are solely those of the authors and do not necessarily represent those of their affiliated organizations, or those of the publisher, the editors and the reviewers. Any product that may be evaluated in this article, or claim that may be made by its manufacturer, is not guaranteed or endorsed by the publisher.
